# Optoelectronic Pressure Sensor Based on the Bending Loss of Plastic Optical Fibers Embedded in Stretchable Polydimethylsiloxane

**DOI:** 10.3390/s23063322

**Published:** 2023-03-22

**Authors:** Alberto Alonso Romero, Koffi Novignon Amouzou, Dipankar Sengupta, Camila Aparecida Zimmermann, Andréane Richard-Denis, Jean-Marc Mac-Thiong, Yvan Petit, Jean-Marc Lina, Bora Ung

**Affiliations:** 1 Electrical Engineering Department, École de Technologie Supérieure, 1100 Notre-Dame Street West, Montreal, QC H3C 1K3, Canada; 2 Hôpital du Sacré-Cœur de Montréal, 5400 Gouin Boul. West, Montreal, QC H4J 1C5, Canada; 3 Mechanical Engineering Department, École de Technologie Supérieure, 1100 Notre-Dame Street West, Montreal, QC H3C 1K3, Canada

**Keywords:** flexible pressure sensor, plastic optical fiber, polydimethylsiloxane, bending, light intensity, commercial electronic components, simple fabrication, cost-effective

## Abstract

We report the design and testing of a sensor pad based on optical and flexible materials for the development of pressure monitoring devices. This project aims to create a flexible and low-cost pressure sensor based on a two-dimensional grid of plastic optical fibers embedded in a pad of flexible and stretchable polydimethylsiloxane (PDMS). The opposite ends of each fiber are connected to an LED and a photodiode, respectively, to excite and measure light intensity changes due to the local bending of the pressure points on the PDMS pad. Tests were performed in order to study the sensitivity and repeatability of the designed flexible pressure sensor.

## 1. Introduction

The quick advancement of science and technology in the fields of artificial intelligence, the Internet of Things, smart devices, new materials, power supplies, sensing modalities, and assembly techniques is providing impetus for the development of new flexible skin-like sensors based on flexible electronics [1,2,3]. Electronic devices that can bend, stretch, and fit curved surfaces without losing functionality are known as flexible electronic sensors [4,5,6,7]. These sensors can be attached to the human body (externally or internally) [3,8] or civil structures [9,10] for a variety of promising applications in healthcare, biomedicine [8], human–machine interfaces, soft robotics, sports performance, wearable electronics [3,4], structural health monitoring, security, and environmental monitoring [9,10,11]. Their ability to conform to surfaces by removing device motion or mechanical mismatch [2,8,12] enables continuous, dynamic, and accurate assessment of a variety of physiological parameters (pulse rate, body temperature, gait analysis, heart rate, sleep quality assessment [1,5,7,13], tactile perception [5,6], pressure monitoring at pressure points in bedridden patients [14,15], detection of pressure areas in wheelchair patients [16], muscle activity monitoring [1,6,13], among others) or the detection of stress, cracks [9,17], or damage in bigger structures such as airplanes, bridges, buildings, or other civil constructions [10,11,18].

These adaptable sensors are made of flexible materials and can be manufactured in a variety of shapes and sizes to detect various parameters, such as pressure, through various sensing principles [13,19], such as electrical resistance [16,20,21], capacitance [20,22], piezo-electricity [23], resonance [11], or fluctuations in light intensity [24,25]. In contrast to rigid electronic devices, which have stricter shape limitations [26], higher risk of mechanical failure (particularly when subjected to strain or deformation that is incompatible with their rigid structure) [27], more difficult integration with tissues or organic materials [28], and higher weight and volume [8,27], flexible electronics can combine a variety of electronic components with flexible material hosts that can withstand a wide range of strains, such as tension, compression, bending, or torsion [3,17], with significant benefits including design flexibility, lightness and thinness, manufacturing versatility, and cost-effectiveness [26,29,30]. These sensing devices must also comply with specific requirements, such as being bio-compatible [12,31], safe [3,12,31], lightweight, non-toxic [3], stretchable, flexible [3,12], and hydrophobic [8,17], to ensure that they are tightly integrated and adhered to. Furthermore, as wearable health monitoring devices or human–machine interaction interfaces, their design should enable them to sustain sensing performance throughout long periods of use and extensive usage cycles [3,9,12,17].

Several materials have been investigated to address the challenge of fabricating flexible sensors that allow for repeated application at maximum strain, including polyethylene terephthalate (PET), polyethylene (PEN), polyurethane (PU), polyimide (PI), polycarbonate (PC), polydimethylsiloxane (PDMS), hydrogels, and cellulose fibers. PDMS is a promising and effective host material for optoelectronics due to its outstanding deformability and ease of production [3,8,9,32]. The incorporation of optical fibers (OFs) into the host material (PDMS) could aid in the creation of flexible OF-based sensors. Optical pressure sensors, which detect variations in the intensity of light passing through or reflected from a pressure-sensitive material to measure pressure, are the most well-known OF-based sensors. These sensors use optical sensing techniques such as total internal reflection, absorption, and light emission [11,13,24,33]. The most common optical pressure sensing approach is one based on mechanical deformation that changes the amount of light traveling through the optical fiber [10,34,35]. This method is based on the idea that when an optical fiber is bent or curved, light is deflected, and some energy is lost due to dispersion. The amount of light lost is proportional to the sensor’s curvature or bending. This change in light intensity can be measured with a photodetector, which allows the amount of pressure exerted to be calculated [30,36]. Sensors based on this technology have some advantages over rigid electronic sensors, such as electromagnetic immunity, corrosion resistance, electrical isolation, environmental resistance, compactness, being lightweight, and high sensitivity [33,34,35], which makes them suitable for industrial, healthcare, and bio-medical research. In this paper, we propose combining plastic optical fibers into a flexible and elastic PDMS host to build a two-dimensional flexible and stretchable sensing pad.

## 2. Materials and Methods

### 2.1. Sensor Pad Manufacturing Process

The flexible sensor samples work under the sensing technique of optical intensity modulation, in which the actuation (via pressure) of the crossing or intersection point of fibers results in optical fiber bending loss. To study the working principle of the sensor and the performance of the selected electronic devices, two samples with different numbers of OFs were designed. The first sample consists of two PMMA plastic optical fibers (POFs) encapsulated in a PDMS pad, with both fibers having a diameter of 0.25 mm (Mitsubishi Chemical Super Eska™ Optical Fiber, SK-10; Industrial Fiber Optics, Inc., Tempe, AZ, USA) and a length of ~120 cm. To induce larger optical losses, one of the fibers was crossed perpendicularly on top of the other identical fiber to create a single pressure point (PP) at the intersection of both fibers (A1). For the second sample, the design consists of 4 such optical fibers in a grid array within a PDMS pad, with the fibers parallel to each other and spaced by 1 cm. Two of the four POFs are crossed perpendicularly on top of the other two fibers, creating a two-row (A and B) by two-column (1 and 2) arrangement of POFs. The intersection of each row and each column is related to a local PP, giving a total of 4 pressure points (A1, A2, B1, B2). For both samples, care was taken during assembly of the intersection of fibers so that the columns (1 and 2) were always above (Up) and the rows (A and B) always below (Dwn) in the crossing.

**Figure 1:**
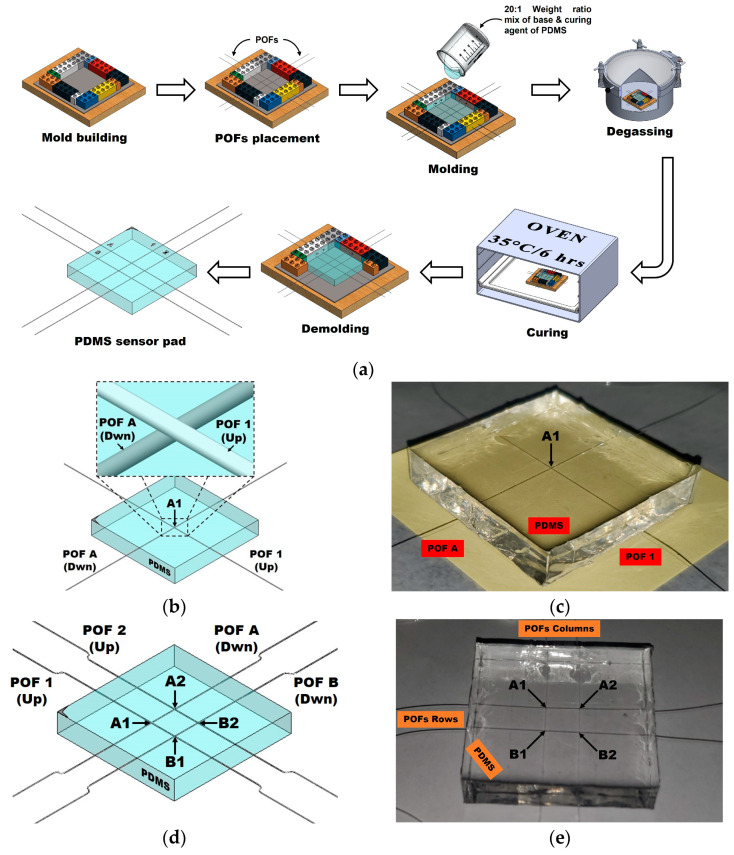
(**a**) Graphical representation of the manufacturing process of molding the sensor pads; (**b**) 3D model of the sample of 1 POF × 1 POF and the pressure point (A1) resulting from the intersection of the fibers; (**c**) real photo of the 1 POF × 1 POF sample and its pressure point A1; (**d**) 3D model of the 2 POFs × 2 POFs sample and its pressure points resulting from intersection of the fibers (A1, A2, B1, B2); (**e**) real photo of the 2 POFs × 2 POFs sample and its pressure points (A1, A2, B1, B2).

To mold the PDMS into pads, LEGO^®^ blocks were used to create square molds of 5 cm × 5 cm, in which precise holes were drilled on each side to allow the POFs to pass through according to the crossing order mentioned above. The fibers were then encapsulated in a 20:1 weight ratio PDMS (Dow Corning, Sylgard™ 184; Dow Corporate, Midland, MI, USA) base and curing agent mixture that was mixed for about 3 min using a magnetic stirrer. This ratio provides a good trade-off between the hardness and elasticity of PDMS for the purposes of this experiment and keeps the intersections of fibers in their positions. When the mixture was ready, it was poured into the mold to completely cover the fibers, thereby creating 5 cm × 5 cm × 0.8 cm pads. The PDMS pads were degassed for one hour in a vacuum chamber to eliminate microscopic air bubbles in order to ensure good material homogeneity. After degassing, the PDMS mold was left to cure at 35 °C for six hours, after which the pads were finally extracted via demolding once they had cooled to room temperature. The manufacturing process of the pads, their three-dimensional representation, and images of actual samples are shown in Figure 1.

### 2.2. Low-Cost Electronics Design

**Figure 2:**
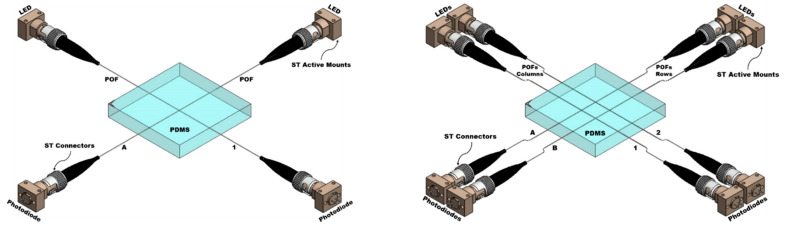
Three-dimensional models of the 1 POF × 1 POF and 2 POFs × 2 POFs sensor pads with the light sources (LEDs) and photodiodes coupled to each fiber.

In order to complete the optical fiber sensors’ elements, low-powered and compact opto-electronics components were selected with the goal of favoring cost-effective integration into flexible sensor devices. Red LEDs (Marktech Optoelectronics, MTE7063NK2-UR; Marktech Optoelectronics, Inc., Latham, NY, USA) with a wavelength of 630 nm were coupled to one end of each fiber as a light source, while a photodiode (Marktech Optoelectronics, MTD3910N; Marktech Optoelectronics, Inc., Latham, NY, USA) with a response range of 400 to 1060 nm was coupled to the other fiber end in order to measure the change in light intensity through the fibers. ST active device mounts and ST connectors are used to couple light from LEDs going in to the optical fibers at the input with the photodiodes at the output, as shown in Figure 2.

**Figure 3:**
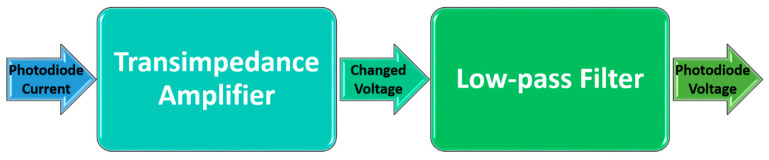
Block diagram of the electronic stages designed for the photodiodes of the sensor pads.

A measured light intensity (photodiode response) of a few µA was passed through a transimpedance amplifier (TIA) (Analog Devices, LTC6268; Analog Devices, Inc., Wilmington, MA, USA), which changed the low-level current signal of the photodiode to a usable voltage output (~4.5V). Finally, this voltage was passed through an active low-pass filter of 60 Hz with a gain of 1.5 to remove most of the electronic noise from the signal. The output signal of the whole electronic circuit, which was given the name photodiode voltage, is connected to a multimeter in order to visualize the voltage changes in response to the pressure exerted on the pressure points of the sensor pads (Figure 3).

### 2.3. Experimental Setups

**Figure 4:**
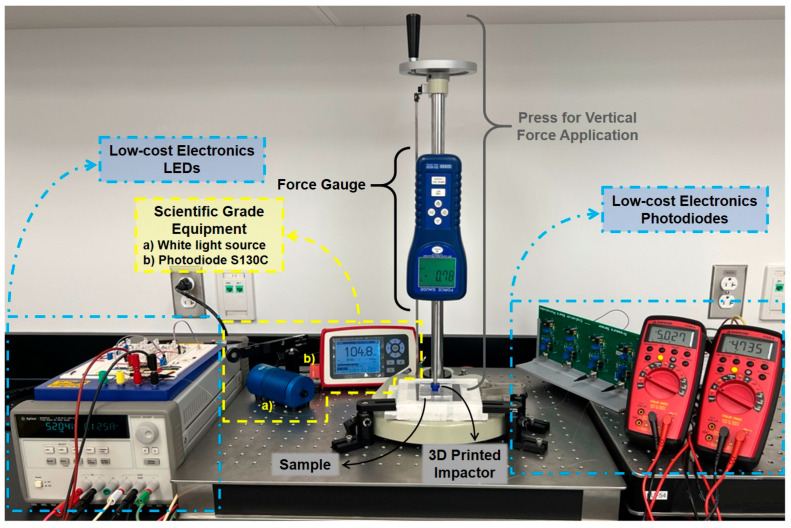
Tools and equipment used in experimental setups.

The main interest is knowing the sensitivity and repeatability of the components selected for the manufacture of this flexible pressure sensor built with low-cost components. For doing that, the samples and the designed electronics were tested under two different configurations: (1) the use of scientific-grade equipment, designed and calibrated to make optical measurements, and (2) the use of a low-cost electronics setup. For each of these configurations, two measurement repetitions were carried out for each sample, in which the pressure points of each sample pad were individually tested by applying a vertical force of 0 N to 40 N with the help of a force gauge (REED Instruments, SD-6020; REED Instruments, Wilmington, NC, USA) and a 1 cm diameter 3D printed impactor. In accordance with the vertical force applied with the force gauge and the area of the circular face of the 3D printed impactor, the samples were stressed from 0 kPa up to ~509 kPa (Figure 4).

For each test and repetition, the same experimental protocol was followed. This started by placing the sensor pad on a metal plate and manually placing each PP under the impactor on the force gauge tip. The impactor was then placed so that it lightly touched the top face of the pad without applying any pressure (0 kPa), and a light and photodetector stabilization period was used to obtain the initial value of light intensity (voltage). The pressure was then increased to ~63 kPa (or 5 N of applied force), and after a stabilization period of a few seconds, which is when the measurement devices give a steady reading, the new light intensity value (voltage change) was recorded. Later, the pressure was increased to ~127 kPa (10 N) and the new light intensity value was recorded. After this point, the pressure increments were made every ~127 kPa until ~509 kPa was reached. When the last pressure was reached, the output light intensity was recorded and it was withdrawn, with a rest time given for materials to recover their original shape. We then proceeded to perform a second repetition on the same PP following the same steps mentioned above. After the second repetition, the pad was moved to the next PP and everything was repeated.

**Figure 5:**
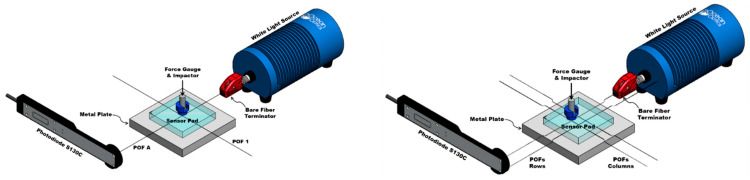
Three-dimensional representation of the experimental setups used to expose the 1 POF × 1 POF (**left**) and 2 POFs × 2 POFs (**right**) samples to the pressures selected with the scientific equipment.

For the benchmark measurements with scientific-grade equipment, once the pad was placed in the described setup, the scientific-grade equipment, a halogen white light source (Ocean Optics, HL-2000-HP Light Source; Ocean Optics (Ocean Insight), Orlando, FL, USA), and a calibrated photodiode (Thorlabs, Photodiode S130C; Thorlabs Inc., Newton, NJ, USA) were attached to each end of a fiber. The HL-2000-HP light source was coupled to the POFs using a Thorlabs’ Universal Bare Fiber Terminator (Thorlabs, BFT1; Thorlabs Inc., Newton, NJ, USA), and the S130C photodiode was placed opposite and as close as possible to the other end of the fiber without any connector or coupling, as shown in Figure 5.

**Figure 6:**
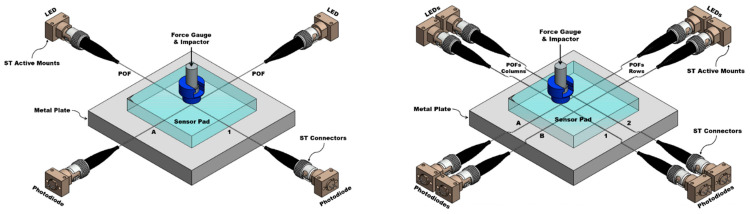
Three-dimensional representation of the experimental setups used to expose the 1 POF × 1 POF (**left**) and 2 POFs × 2 POFs (**right**) samples to the pressures selected with the LEDs and photodiodes coupled to each fiber.

For the characterization of low-cost electronics, after testing the samples with the scientific-grade equipment specifically designed for optical measurements, the previously used equipment was replaced with the designed low-cost electronics. To obtain the peak emission wavelength of the LED, the supply voltage was regulated to obtain a current of 20 mA using a variable trimpot resistor. Similar to the previous setup, all visible light LEDs, photodiodes, and optical fibers were coupled using ST active mounts and ST connectors. The parameters of the electronic amplifier and filter circuits were adjusted by their variable gain resistors so that the output would give an approximate value of 5 V. Following the described experimental protocol, each PP was stressed at the selected pressures, and each new photodiode voltage was recorded manually (Figure 6).

## 3. Results

### 3.1. Benchmark Measurements with Scientific-Grade Equipment

**Figure 7:**
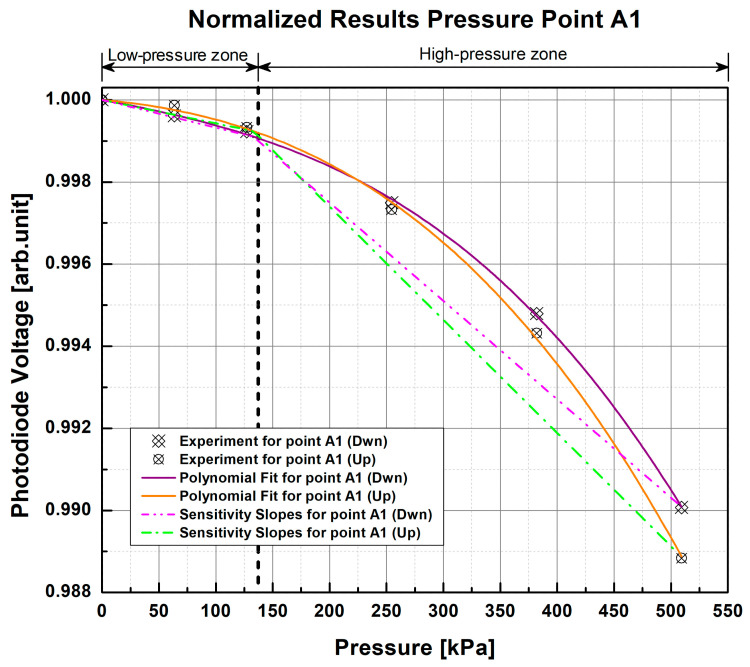
Normalized response curves of the 1 POF × 1 POF sample with the scientific-grade equipment setup for pressure point A1. The experimental data points correspond to the average of two test repetitions.

**Figure 8:**
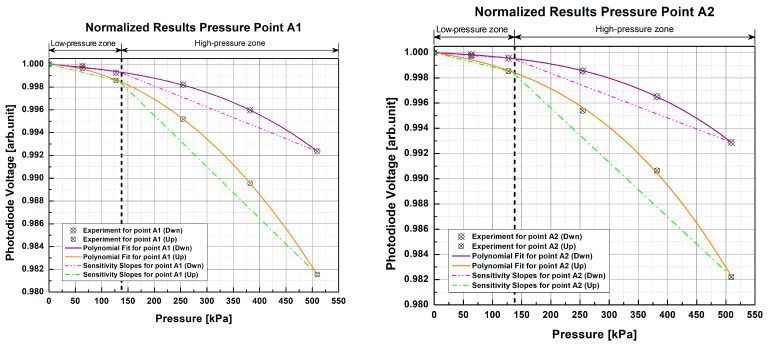

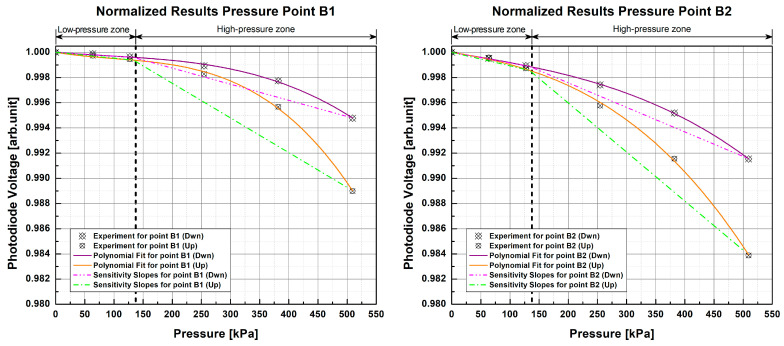
Normalized response curves of the 2 POFs × 2 POFs sample with the scientific-grade equipment setup at different pressure points. The experimental data points correspond to the average of two test repetitions.

The results obtained from this test will serve as a reference for the repeatability and sensitivity of the samples to different values of applied pressure based on the measurements obtained with scientific-grade equipment. Following the experimental protocol described above, each PP was subjected to the selected range of pressure values, and corresponding changes in the photodiode voltage from the S130C photodiode were recorded. Using this equipment, the reference response curves of each PP of each sample were obtained (Figure 7 and Figure 8), in which a non-linear and repetitive response can be observed in each measurement, indicating that the higher the pressure exerted, the lower the light intensity at the exit.

In the non-linear response of both samples, it can be observed that they begin to show small changes around 63 kPa to 127 kPa, while there is greater light attenuation from ~127 kPa to ~509 kPa. For this reason, two pressure zones were defined for the measurements according to the graphical perception of changes in photodiode voltage; therefore, there is a low-pressure zone from 0 kPa to ~133 kPa and a high-pressure zone from ~133 kPa to 550 kPa to help with analysis of the results. As can be seen in the graphs, the changes in voltage are more pronounced in the high-pressure zone as pressure increases, which would indicate that the samples are more sensitive to higher pressures. In contrast, in the low-pressure zone (below 133 kPa), the light intensity changes have a smaller magnitude than those of the high-pressure zone, which may indicate that the tested sample cannot distinguish pressure changes below this threshold.

**Figure 9:**
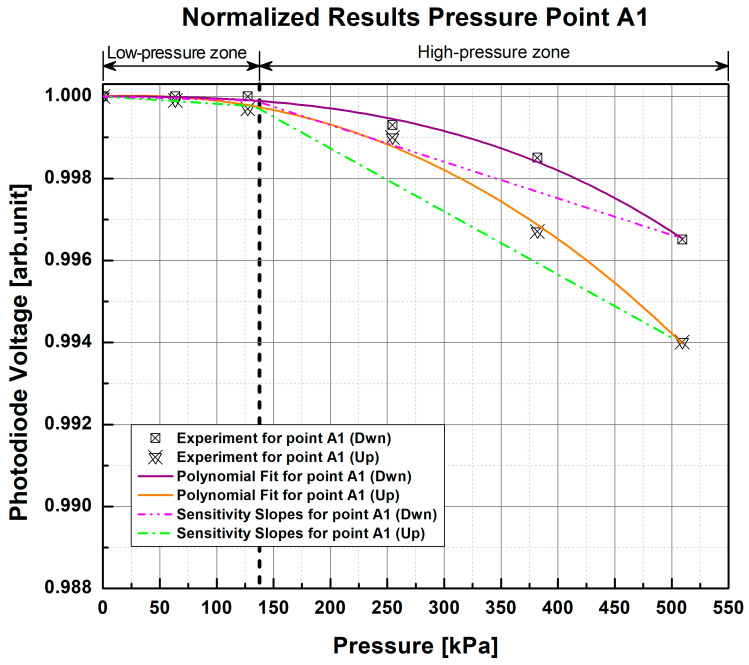
Normalized response curves of pressure point A1 for the 1 POF × 1 POF sample with the low-cost electronics setup. The experimental data points correspond to the average of two test repetitions.

**Figure 10:**
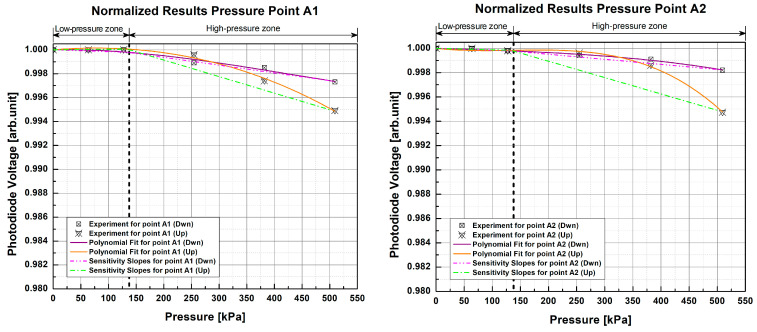

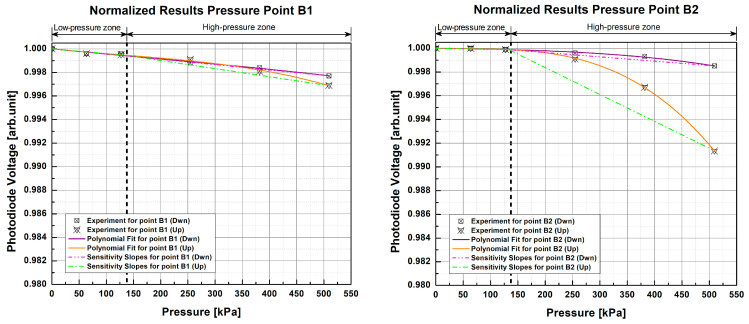
Normalized response curves of the 2 POFs × 2 POFs sample with the low-cost electronics setup at different pressure points. The experimental data points correspond to the average of two test repetitions.

In order to verify this, the average of the results obtained from each repetition of each fiber was calculated. Polynomial fit was obtained by the polynomial functions

(1) $$f\left(x\right)=a_{n}x^{n}+a_{n-1}x^{n-1}+\cdots +a_{2}x^{2}+a_{1}x+a_{0}$$

that best define the response of each fiber. The function that best describes the average of each pair of repetitions of every fiber was a third-order function (*n* = 3), where the coefficients $a_{3}$, $a_{2}$, and $a_{1}$ were calculated and the coefficient $a_{0}$ restricted to 1 ($a_{0}=1$) by the normalization of results. The third-order functions that characterize these curves have a goodness of fit (GOF) of $R^{2}=0.9995$, $R_{adj}^{2}=0.9992$, and $RMSE=1.1301\times 10^{-4}$ and were used to obtain photodiode voltage in the low- and high-pressure zones defined in order to calculate the average slope of each zone (dash-dot and dash-dot-dot lines in Figure 7, Figure 8, Figure 9 and Figure 10). In general, for the 1 POF × 1 POF sample, it was found that the low-pressure zone had an average slope of $-6.227\times 10^{-6}\;arb.unit/kPa$, while the high-pressure zone had a slope of $-2.576\times 10^{-5}\;arb.unit/kPa$. On the other hand, the 2 POFs × 2 POFs sample had an average slope of $-7.221\times 10^{-6}\;arb.unit/kPa$ for the low-pressure zone and $-2.790\times 10^{-5}\;arb.unit/kPa$ for the high-pressure zone. Comparing these results, it can be seen that the samples did indeed have greater sensitivity to high pressures owing to their steeper slope. Similarly, it can be observed in all the graphs that, despite being in the same PP, the fibers at the top (Up) of the fiber crossing of each pressure point were more sensitive than the one lying at the bottom (Dwn) because they suffered from greater attenuation of light due to fiber bending loss.

By evaluating the relative standard deviation (RSD) of the results obtained for each value of applied pressure, we can see that the 1 POF × 1 POF sample has, on average, an RSD=0.0427%, while for the other sample (2 POFs × 2 POFs), the RSD value is equal to 0.0280%. The low RSD percentage indicates high measurement precision, i.e., low dispersion between each measurement and the average of the repetitions.

### 3.2. Characterization of Setup Using Low-Cost Electronics

After testing the samples with scientific-grade equipment, this was replaced with the designed low-cost electronics. This test helped to investigate whether the selected photodiode and the designed electronics are sensitive enough to detect small optical signal changes when pressing the PPs.

The normalized response curves of each sample for this test (Figure 9 and Figure 10) show a non-linear and repetitive behavior very similar to that of the previous test, albeit with a smaller amplitude. In comparison with the previous test, it can be seen that within the low-pressure area, the light changes are minimal and begin to be appreciated, in most cases, from ~127 kPa onwards. Furthermore, light intensity varied within the range of 0.980 arb.unit to 1 arb.unit with the scientific-grade equipment, while for this test using low-cost components it was within the range of 0.990 arb.unit to 1 arb.unit, making the response curves flatter than the previous ones; however, they still show a more significant attenuation in light intensity as pressure increases and enters the high-pressure zone. This suggests that the samples tested with the low-cost electronics also could not distinguish pressure changes below the 133 kPa threshold. This reduced magnitude or flattening of the curves results in a loss of sensitivity when using the low-cost electronics. Further, as in the previous test, the slopes in each pressure zone were calculated to see how the sensitivity of the pads was affected by the change in light source and photodetector.

In the low-pressure zone, the photodiode voltage changes were very small, and, after having calculated the slopes with the third-order functions (1) with a GOF of $R^{2}=0.9934$, $R_{adj}^{2}=0.9891$, and $RMSE=1.3987\times 10^{-4}$, it is noticeable that the 1 POF × 1 POF sample has an average slope of $-1.275\times 10^{-6}\;arb.unit/kPa$, and that the 2 POFs × 2 POFs sample has an average slope of $-1.735\times 10^{-6}\;arb.unit/kPa$, both of which are lower than the values obtained for the low-pressure zone in the test with scientific-grade equipment. Similarly, for the high-pressure zone, slopes of $-1.216\times 10^{-5}\;arb.unit/kPa$ and $-9.464\times 10^{-6}\;arb.unit/kPa$ were obtained for the 1 POF × 1 POF and 2 POFs × 2 POFs samples, respectively, presenting greater slopes than those of the low-pressure zone and reinforcing that the samples studied are more sensitive to high pressures. However, these slopes are not as steep as those obtained for this pressure zone in the reference test, confirming that there is a loss of sensitivity with low-cost electronic components. A comparison shows that there is an average loss of 66.1659% in sensitivity for the 1 POF × 1 POF sample with the low-cost electronics, which is 71.0284% for the 2 POFs × 2 POFs sample when compared to the reference scientific setup.

Despite having lower sensitivity than the scientific-grade configuration, it can be seen that the pads keep their sensitivity at higher pressures by having a steeper slope than the low-pressure zone, making the attenuation of light intensity greater as the pressure increases. Additionally, the low-cost electronics designed to detect light changes when the fibers of each PP are pressed present a non-linear and repetitive behavior similar to that of the scientific equipment, although not as severe. As in the previous case, it can be seen that the fiber at the top (Up) of the crossing in most cases exhibits higher optical attenuation and therefore greater sensitivity than the fiber located just below (Dwn).

The RSD of the results of the samples with the designed low-cost electronics is 0.0270% for the 1 POF × 1 POF sample and 0.0368% for the 2 POFs × 2 POFs sample. As in the previous test, low RSD indicates high measurement repeatability. Comparing these results with those of the reference test, we can see for the 1 POF × 1 POF sample that the RSD of the low-cost electronics setup is 0.0157% lower than that of the scientific-grade equipment. This comparison could appear to suggest that low-cost electronics present higher measurement precision than specialized equipment; however, this is not so, since we can see in the low-pressure zone (Figure 9) that the results of both repetitions are minimal. There is no change at all here, and you can even go so far as to say that the samples were insensitive in this zone. This makes evaluations of RSD come out with a lower average value since the results without change (SD = 0) are left out of the calculations. Similarly, when comparing the results of the second sample, it is observed that the low-cost electronics setup had a higher RSD and that the difference with the scientific equipment was 0.0088%, which indicates that there is some loss of precision. Likewise, the slight difference between both dispersions is because the changes in the low-pressure zones (Figure 10) of each pressure point are minimal or nonexistent with the electronic elements, which causes some of them to be left out (SD = 0) of the calculations, thus resulting in lower RSD.

**Figure 11:**
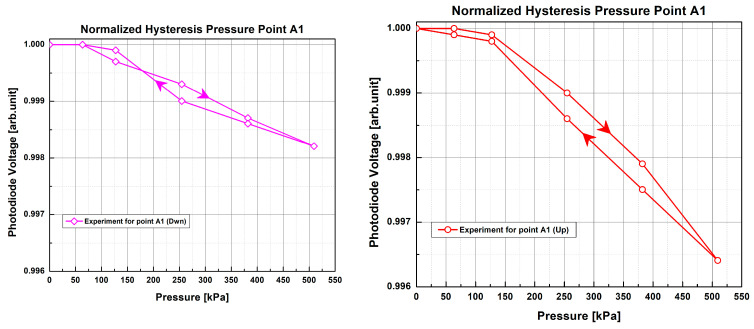
Hysteresis curves of the 1 POF × 1 POF sample with the low-cost electronics setup at pressure point A1. The experimental data points correspond to the average of two test repetitions. The arrows indicate the direction of applied pressure (i.e. downward and then upward).

Finally, in Figure 11, we note the difference between light intensity values when the applied pressure was gradually and continuously applied upward and then reversed at PP A1 of the 1 POF × 1 POF sample with the low-cost electronics setup. Since the sensor is made of flexible and stretchable materials, we observe that the response curves (Figure 11) present some small hysteresis behavior, with curves overlapping in their maximum and minimum endpoints. Because PMMA and PDMS are both amorphous and viscoelastic materials, the observed small hysteresis may be attributed to a combination of chain relaxation, changes in free volume [37], and stress softening [38]. Stress softening is believed to be a primary factor for hysteresis in elastomers such as PDMS [38]. This has been observed in silica-filled PDMS, such as Sylgard 184, and is thought to arise from a decrease in filler–PDMS chain interaction and chain entanglement [39,40]. It can also be seen in these curves that, to reach minimum light intensity in the range of evaluated pressures, the same amount of pressure is required, irrespective of whether the fiber is placed on the top (Up) or bottom (Dwn) position.

Generally speaking, the purpose of these tests was to gradually compare the functionality, precision, repeatability, and performance of the low-cost designed elements against scientific-grade equipment. Comparing the normalized response curves of each test, it can be observed that the response of the electronic components presents a non-linear and repetitive behavior similar to that of the scientific equipment, in which photodiode voltage decreases as pressure increases. It can be seen in the results graphs that the greatest changes in light intensity were generated mainly in the high-pressure zones, i.e., above 133 kPa, which indicates that the materials are more compressed and deformed above this pressure. In all our tests, we were able to deform the sensor pads by over 40% compared to their initial uncompressed state without causing permanent damage. All samples were also able to recover their initial state after testing. The higher sensitivity of samples above this pressure may be due to the fact that the thickness of the pad prevents the fibers from sensing the lower pressure values since there is no significant deformation or bending of the underlying POF.

It was to be expected that the low-cost electronics would experience a loss of sensitivity compared with the scientific-grade equipment. Some of the factors that could explain this loss of sensitivity would be the large coupling losses (>30 dB) observed between the LED light sources and the photodiode detectors. This meant that the low power incident on the photodiodes generated a very low current value (~1.4 µA). Additionally, even though the TIA used had the needed characteristics to amplify these low currents, a very high gain value had to be implemented, which, in some cases, reached the electronic limits of the TIA. Another factor that could have affected the results is the low number of repetitions for each PP since only two were made with each sensitive pad in each test. A higher number of repetitions will give more results, which will increase the dispersion between them; consequently, the RSD will vary and will be able to give an updated precision value.

Another factor that could have influenced the results is the manufacturing processes of the optical fiber supplier. Although the fiber samples used for the sensor came from the same manufacturing batch, the diameters of the core and cladding varied slightly from length to length, causing their diameter to be non-uniform. Such small differences can create discrepancies when assembling the grid array of fibers for the pads. For example, discrepancies in dimensions between the top and bottom fibers can prevent them from remaining in contact after PDMS is poured into the mold, thus creating an imperfect contact point between the fibers that prevents them from properly deforming when pressed upon. We believe that this is what happened in the measurements from point B1 of the 2 POFs × 2 POFs sample (Figure 8 and Figure 10) since, as can be noted in the graphs, the changes in light intensity are significantly smaller (approximately 45% loss in sensitivity) compared to the other pressure points. This indicates that the current manual and labor-intensive manufacturing process is prone to errors.

Despite this, the results obtained help to conclude that the selected electronic components fulfilled their functions as light sources and photodetectors since the fibers were kept illuminated at all times and their light intensity changes were detected; these changes were mainly detected at high pressures, starting at 133 kPa, exhibiting good repeatability despite having lower sensitivity and precision.

## 4. Conclusions

This paper describes the simple fabrication and proof-of-concept demonstration of a soft materials-based pressure sensor embedded with a network of plastic optical fibers. Custom molds were used to encapsulate a number of fibers into a single-layer configuration of polydimethylsiloxane (PDMS) host material that was square pad-shaped. Commercial LED sources and photodiodes were used in an intensity modulation scheme for monitoring the pressure values on the flexible pad. Based on the good repeatability and wide dynamic measurement range obtained with this configuration, the implementation of this type of sensor could benefit industrial applications (e.g., industrial robotics, manufacturing, or structural health monitoring) where a large range of pressures (0 to 550 kPa) must be monitored. This demonstration was limited in terms of the absolute sensitivity achieved ($<10^{-5}\;arb.unit/k\mathrm{Pa}$), which remained low due to the strong hardness of the PMMA fibers used in this work. A promising approach to explore in the future to improve the sensitivity of this type of sensor is to use softer plastic fibers such as elastomers. This work represents another step towards the integration of flexible sensing optical fibers within stretchable material hosts which will allow for the fabrication of flexible, accurate, conformable, and cost-effective sensors.

## Data Availability

Data are contained within the article.
